# Estimating progression-free survival in patients with glioblastoma using routinely collected data

**DOI:** 10.1007/s11060-017-2619-1

**Published:** 2017-09-27

**Authors:** Charlotte Kelly, Paulina Majewska, Stefanos Ioannidis, Muhammad Hasan Raza, Matt Williams

**Affiliations:** 1 Department of Clinical Oncology, Charing Cross Hospital, Fulham Palace Rd., London, W6 8RF UK; 2 School of Medicine, Imperial College London, Exhibition Road, London, SW72AZ UK; 3 Department of Neurosurgery, Charing Cross Hospital, Fulham Palace Rd., London, W6 8RF UK; 4 Computational Oncology Group, Institute for Global Health Improvement, Imperial College London, Exhibition Road, London, SW72AZ UK

**Keywords:** Glioblastoma, Routine data, Overall survival, Progression free survival

## Abstract

Glioblastoma (GBM) represents 80% of all primary malignant brain tumours in adults. Prognosis is poor, and there is a clear correlation between disease progression and deterioration in functional status. In this pilot study we assess whether we can estimate disease progression and progression free survival (PFS) from routinely collected electronic healthcare data. We identified fifty patients with glioblastoma who had chemo-radiotherapy. For each patient we manually collected a reference data set recording demographics, surgery, radiotherapy, chemotherapy, follow-up and death. We also obtained an electronic routine data set for each patient by combining local data on chemotherapy/radiotherapy and hospital admissions. We calculated overall survival (OS) and PFS using the reference data set, and estimated them using the routine data sets using two different methods, and compared the estimated measures with the reference measures. Overall survival was 68% at 1 year and median OS was 12.8 months. The routine data correctly identified progressive disease in 37 of 40 patients and stable disease in 7 of 10 patients. PFS was 7.4 months and the estimated PFS using routine data was 9.1 and 7.8 months with methods 1 and 2 respectively. There was acceptable agreement between reference and routine data in 49 of 50 patients for OS and 35 of 50 patients for PFS. The event of progression, subsequent treatment and OS are well estimated using our approach, but PFS estimation is less accurate. Our approach could refine our understanding of the disease course and allow us to report PFS, OS and treatment nationally.

**Electronic supplementary material:**

The online version of this article (doi:10.1007/s11060-017-2619-1) contains supplementary material, which is available to authorized users.

## Introduction

Glioblastoma (WHO grade IV glioma; GBM) is a rare tumour with 79,000 new cases worldwide per year. However, it represents 1/3rd of primary brain tumours, and 80% of all primary malignant brain tumours in adults [1]. Survival is poor, and even with aggressive treatment the median survival is 15 months. Although life expectancy is important, many patients also want to know how long it will be until they are less well. The development of progressive disease is an important event for patients and their families, and there is a clear correlation between disease progression and deterioration of both functional status and quality of life [2, 3]. The anatomical location of a glioblastoma within the central nervous system leads to different clinical presentation of neurological impairments of varying degrees. Therefore functional status and subsequently quality of life is dependent on the neurological impairment at presentation and disease progression. Clinical trials provide the best quality data on treatment and progression but less than 10% of patients are enrolled in clinical trials, and those who are enrolled differ from those treated in routine care [4].

To measure outcomes in routine practice, prospectively collected manual data (as in clinical trials) remains the gold standard. However, this is time consuming and expensive, and there is increasing interest in the use of routinely collected electronic health data [5]. Such data is typically generated as a by-product of care, such as prescriptions and billing, and have the advantage of being available at a large scale and for relatively little cost when compared to dedicated multi-centre manual data collection. Although we can measure overall survival (OS) from routinely collected data [6], the challenge remains to estimate disease progression, and progression-free survival (PFS), from these data sets. The ability to do this would allow us to assess these outcomes, which are of significant relevance to patients, at a national level relatively quickly and cheaply. We have previously used electronic data to estimate disease progression in patients with head & neck cancers [7, 8], and others have used similar approaches in breast cancer [9]. However, there has been no work in patients with brain tumours.

In this pilot study, we extend our previous work to patients with glioblastoma receiving chemo-radiotherapy. We assess whether we can estimate rates of progression and PFS using routinely collected electronic data, and compare that estimate with the actual rate of progression and PFS based on manually curated patient data.

## Method

We identified a pilot group of 50 patients with histologically confirmed glioblastoma (WHO grade IV glioma) who received chemo-radiotherapy in a single center under the care of a single treating consultant oncologist. Data collection was completed on 30th April 2016.

The standard practice for patients with glioblastoma receiving treatment in our center consists of neurosurgical tissue sampling (with surgical resection where possible), followed by chemo-radiotherapy (60 Gy in 30 fractions over 6 weeks) with concurrent chemotherapy (temozolomide 75 mg/m^2^). Patients undergo an MRI scan 3 weeks after the end of chemo-radiotherapy, and then receive 6 or 12 cycles of adjuvant temozolomide (150–200 mg/m^2^), depending on patient choice. This is identical to the regimen used in the randomized phase III trial [10], but with the consideration of delivery of an additional six cycles of temozolomide. Patients who have imaging suggestive of progression on their scan post chemo-radiotherapy are assessed clinically. If well, they continue with temozolomide and have a repeat MRI scan after 2 (rather than 3) cycles of chemotherapy. If sequential MRIs show deterioration in appearances, we regard the initial MRI as showing true (rather than pseudo) progression. Progression is confirmed on serial MRI, using RANO criteria, with all scans reviewed by an experienced neuro-radiologist.

### Reference data

**Fig. 1:**
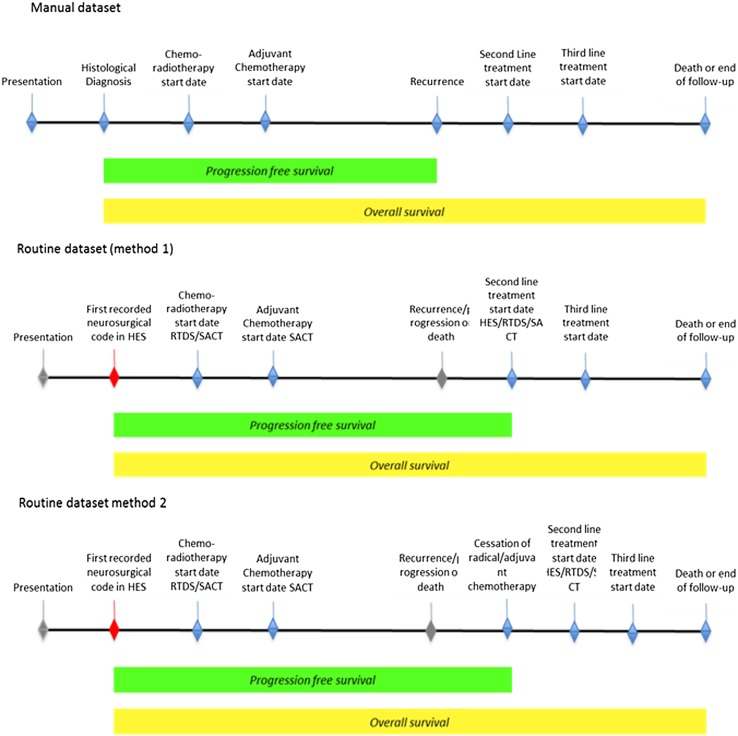
Relative time points in the manual and routine dataset

For each patient, we manually extracted a reference data set from multiple sources (clinic letters, multidisciplinary meeting outcome proformas, pathology results system, discharge summaries, radiotherapy and chemotherapy treatment summaries and hospital notes) as a summary of their diagnosis, treatment and outcomes. This was our reference data set. Overall survival was the interval from the date of histological diagnosis to date of death or last follow-up. Progression- free survival was the interval from histological diagnosis to the first radiological evidence of disease progression (on MRI), or date of last follow-up or death, whichever was earlier (Fig. 1).

### Routine data

For each patient, we also obtained a routine data set by combining local data on inpatient care, radiotherapy and chemotherapy (see Appendix). We extracted dates of hospital admissions, diagnoses and surgical procedures, chemotherapy regimens, dates, number of cycles and duration of treatment and radiotherapy dates, duration of treatment, dose and fractionation. We added data on deaths from the national personal demographics service. The date of last follow-up was obtained from the electronic hospital records system, and date of death from NHS personal demographics service.

Routine data sources do not explicitly contain date of diagnosis or progression. We therefore used clinical events in the routine data to estimate these. Progression was estimated using two methods; method 1 used the start of second line treatment (further neurosurgical resection, administration of second line chemotherapy or re-irradiation) as evidence of progression. Method 2 used either start of second line treatment (as method 1) or cessation of first line treatment as a marker of progression (Fig. 1). Concurrent or adjuvant chemo- or radiotherapy was not considered to be evidence of recurrence. Progression-free survival was estimated as the interval between the date of diagnosis and the estimated date of progression or death or end of follow-up.

### Outcome measures

The primary outcome measure was per-patient agreement for PFS between the reference and routine data sets. Overall and progression-free survival intervals from the reference and routine data sets were considered to have acceptable agreement if the ratio between the two intervals was between 0.75 and 1.25. Our secondary outcomes were correlation between reference and routine datasets for PFS and OS (using Kendall’s *t* test), and per-patient agreement between the manual and reference datasets for OS. We also calculated the performance of the routine data to detect progression, using a standard 2 × 2 table and associated measures (supplementary table 2 & 3).

### Ethical considerations

Ethical approval was not required for this retrospective study and it was registered as a clinical audit reference number 1900 at Imperial College Healthcare NHS Trust.

## Results

### Patient characteristics

We identified fifty patients. 30 were male, 20 were female, and the median age was 58 (supplementary table 1). All 50 patients received external beam radiotherapy (60 Gy in 30 fractions) with concurrent temozolomide chemotherapy (75 mg/m^2^). All were WHO performance score 0–1. Forty-five patients had de-bulking surgery (29 gross total resection, 16 sub-total resection) and five patients only underwent biopsy.

### Survival and recurrence

Overall survival was 68% at 1 year, median OS was 12.8 months and median PFS was 7.4 months. At the time of analysis 40 of the 50 patients had developed progressive disease and 25 of the fifty patients had died. Of the 40 patients who developed progressive disease, 25 commenced second line treatment. Seventeen of the 25 patients received second line chemotherapy (11 lomustine, 1 carboplatin, 2 metronomic temozolomide, 1 bevacizumab with irinotecan, 1 PCV and 1 phase I clinical trial), 5 had further surgery and 3 had further radiotherapy. Fifteen patients received no second line treatment due to deterioration in clinical status.

Using the routine data, OS was estimated as 66% at 1 year and median OS at 12.9 months (Fig. S1). Using method 1, median PFS was estimated at 9.1 months and using method 2 it was estimated to be 7.8 months (Figs. S2 and S3).

### Data agreement and discrepancies

For 49 of the 50 patients, there was acceptable agreement (i.e. estimated interval 0.75–1.25 of the actual time interval) for OS between the two datasets.

**Fig. 2:**
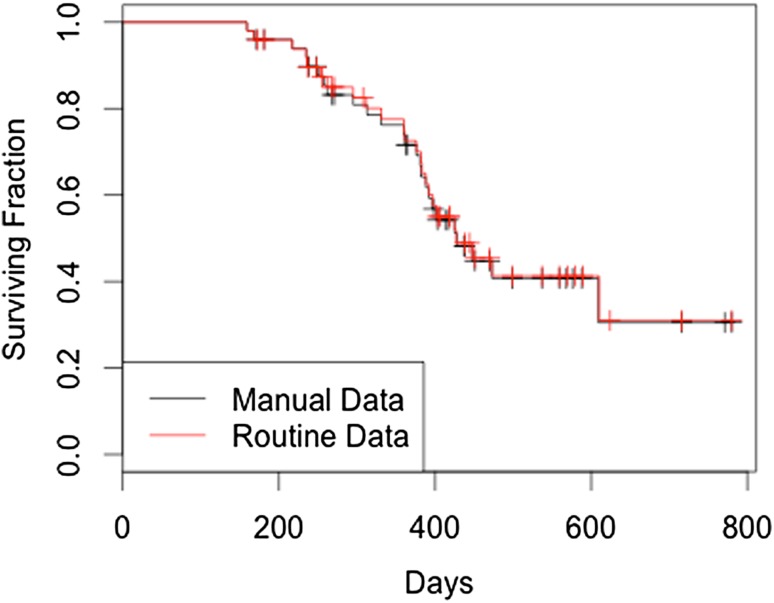
Overall survival in days (by two different methods)

**Fig. 3:**
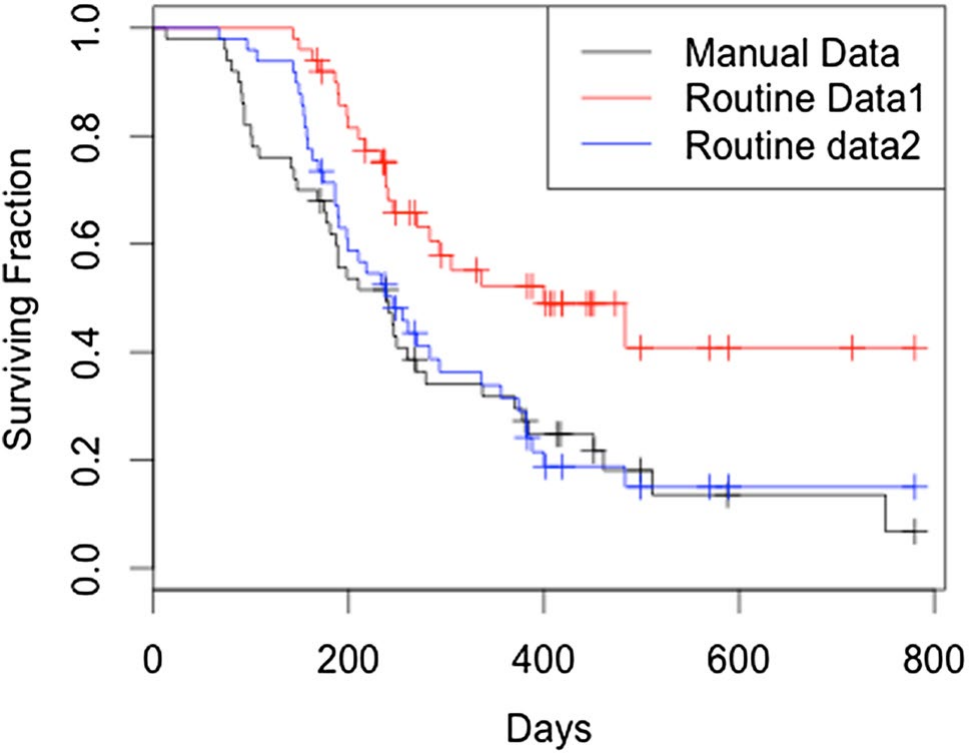
Progression free survival in days (by three different methods)

Of the 40 patients who developed progressive disease, method 1 correctly identified 23 and method 2 correctly identified 37. Of the 23 patients identified with progressive disease by method 1, 15 had an acceptable agreement for estimated PFS with the reference data. Method 2 had an acceptable agreement for 23 of the 37 identified. Of the 10 patients who did not develop progressive disease, method 2 correctly identified 7. Overall survival and progression-free survival curves of manual versus routine data sets are shown in Figs. 2 and 3.

Thirty-one of the 50 patients had acceptable agreement for PFS by method 1 and 35 patients by method 2. The routine data over-estimated the progression-free interval in 14 patients by a median of 3.5 months with method 2 and in 19 patients by a median of 5.4 months with method 1. The routine data underestimated PFS in 1 patient by 8.7 months with method 2.

### Correlation and probability

There was good correlation for OS between the reference and routine data sets (Kendall’s tau = 0.86; p < 0.0001) (Fig. S1). The correlation between the two data sets for PFS was less good (tau = 0.62; p < 0.0001 and 0.70; p < 0.0001 for methods 1 and 2 respectively) (Figs. S2, S3).

The sensitivity that a patient with progressive disease was correctly identified by the routine data is 93% and specificity that a patient without progressive disease was correctly identified is 70%, with a positive predictive value (PPV) of 93% and a negative predictive value (NPV) of 70%.

The probability that a patient who developed progressive disease was both identified and had a reasonable estimate of PFS (“acceptable interval sensitivity”—AISens) was 58%. The AISens and AISpec of method 2 are 58 and 70% respectively, with an AIppv of 88%, and an AInpv of 29% (supplementary table 3).

## Discussion

We have presented a method to estimate disease progression, progression free survival and overall survival from routinely collected data in patients with glioblastoma receiving concurrent chemo-radiotherapy, and evaluated it in a group of 50 patients. Our results are summarised in Table 1. Overall survival and the fact of progression are well estimated using this approach, although PFS estimation is less accurate. The commonest reason for our method to underestimate recurrence was a patient not receiving second-line treatment. The main reason for a discrepancy between actual and estimated PFS was the possibility of pseudo-progression, which as discussed above, can only be determined in retrospect. In theory, our results could be confounded by radionecrosis, but given the low incidence of this (approximately 5% at 1 year) and a median follow-up in our survivors of 13 months, it is unlikely that it significantly influenced our results [11]. Details of the reasons for discrepancies are given in Table 2.

**Table 1:** Identification of progressive and stable disease, and PFS and OS intervals by the manual and routine data sets

Number of patients identified	Progressive disease	Stable disease	OS agreement	PFS agreement
Manual data	40	10	NA	NA
Correctly by routine data (method 1)	23	10	49	15
Incorrectly by routine (method 1)	0	17	1	8
Correctly by routine data (method 2)	37	7	49	23
Incorrectly by routine data (method 2)	3	3	1	14

**Table 2:** Reason for discrepancy between actual and estimated PFS

Reason for disagreement between actual and estimated PFS	Number of patients
Evidence of progressive disease on imaging post chemo-radiotherapy but delay in commencing second line treatment to rule out pseudoprogression	11
Progressive disease post operatively but continued with first line treatment	1
Initiation of second line treatment due to intolerance of first line treatment	1
Cessation of first line treatment due to patient choice	1

Approximately 5% of glioblastoma are secondary, having transformed from a prior low-grade glioma. Unfortunately, both ICD-9 and ICD-10 coding schemes includes grade II, III and IV tumours within the same category. Therefore a patient who had a grade II astrocytoma that transformed into a grade IV tumour some years later, and had surgery for both tumours, would have a discrepancy of years between the manual and routine dataset. This reinforces the importance of using both histology codes and ICD9/10 codes in patients with brain tumours, as opposed to other tumour sites, where ICD codes alone may suffice.

We have assessed the performance of two methods for estimating PFS from routine electronic healthcare data. Method 1 used initiation of second line treatment as evidence of progression. Method 2 used either initiation of second line treatment or cessation of planned first line treatment as markers of progression. As method 2 took account of more of the data, we expected some degree of improvement of our results in reflecting an accurate clinical picture. If we did not incorporate cessation of first line treatment into our methodology we would not detect the patients who clinically deteriorated and stopped first line treatment and would only be detecting progression in patients who were fit for second line treatment. Given the better performance of method 2 we would use this in future work as it more accurately estimates progression. There may be other areas where this approach is applicable, but it depends on being able to define the ‘expected’ clinical course. The extent to which this is feasible is likely to differ across disease types and treatment pathways.

The commonest reason for failing to detect progression was not offering second-line treatment. However, this is heavily influenced by patient fitness, and is likely to be non-uniformly distributed. In principle, we should be able to develop stratified algorithms that have different performance in different subsets of patients, but our pilot study is too small to explore this. In the phase 3 trial demonstrating the benefit of temozolomide, approximately 60% of patients who received chemo-radiotherapy had second-line treatment after progression, comparable to the figures in our cohort.

Previous work in this area has estimated mortality after orthopaedic surgery [12] and oncological treatment [13]. Cancer registry data can be used to measure recurrence rates in breast cancer [9], but there is little work on using routinely available procedure-level data to infer disease recurrence or PFS. One study examined the use of such data to estimate measures of metastatic disease in breast, prostate and lung cancer^,^ while another used patients enrolled in a clinical trial, and we have previously published work on estimating PFS in patients with head and neck cancer [7, 8, 14, 15]. All four studies used a combination of clinical intuition and logically-described criteria to interpret routine data and infer recurrence. Our work is distinguished from these on three grounds. Firstly, this is the first work in patients with brain tumours and secondly, we consider a wider range of interventions (surgery, chemotherapy, radiotherapy) as markers of progressive disease. Finally, we have refined our previous approach to consider both initiation of new treatment and cessation of planned treatment, which improves the estimation of PFS.

Various authors have reported OS from routine data [6, 16] in large national cohorts for patients with aggressive brain tumours, and PFS from clinical trials [17] but there are no reports of progression rates or PFS in large cohorts treated in routine care. We note from the same dataset we have used we can also report the number and length of hospital admissions, number of neurosurgical procedures and total number of treatment lines and time to second progression, and we believe that assessing these non-OS based outcomes needs to become a key part of comparative effectiveness research.

This study pilots our methodology on a group of 50 patients to assess its ability to correctly highlight progression and estimate progression free and overall survival. It directly compares this routinely collected data with a manually collected data set to assess the accuracy of this technique. After validating our method in this pilot we will use the same technique on a national cohort of 4600 patients with glioblastoma, of whom 1500 have received chemo-radiotherapy. This national cohort is composed of patients treated by a range of neuro-oncologists, in a variety of institutions and is comprised of patients who had treatment with radical intent to those who had palliative treatment. By including this cross section of patients we will be able to inform newly diagnosed patients and the families more accurately of their expected disease trajectory.

The approach outlined in this paper is conceptually simple and when applied to large-scale national datasets, such as those reported in the USA [18], Australia [19], France [20] and recently England [6], we should be able to report and compare PFS, OS, treatment and hospital admissions at a national level. This work is currently being planned.

### Electronic supplementary material

Below is the link to the electronic supplementary material.

Figure S1. Manual vs. Routine Overall Survival Interval (TIFF 747 KB)

Figure S2 Manual vs. Routine Progression Free Interval (method 1) (TIFF 747 KB)

Figure S3 Manual vs. Routine Progression Free Interval (method 2) (TIFF 747 KB)

Table 1. Patient Characteristics (DOC 26 KB)

Table 2. Sensitivity, specificity and predictive value (DOC 28 KB)

Table 3. Acceptable interval sensitivity, specificity, and predictive value (DOC 28 KB)

## Appendix

### Sources of data: acronyms and types of data available

National Cancer Registration data (NCRAS): Data on on incidence, demographics and mortality Hospital Episode Statistics (HES): Data on inpatient care—diagnoses and procedures Radiotherapy Dataset (RTDS): Data on radiotherapy—dates, dose and fractionation Systemic Anti-Cancer Therapy dataset (SACT): Chemotherapy—drug and regimen names, dates and doses

### Examples of neurosurgical OPCS codes

**Table:**

	OPCS code	Procedure
Debulk	A021	Excision of lesion of tissue of frontal lobe of brain
	A022	Excision of lesion of tissue of temporal lobe of brain
	A023	Excision of lesion of tissue of parietal lobe of brain
	A024	Excision of lesion of tissue of occipital lobe of brain
	V051	Extirpation of lesion of cranium
Biopsy	A043	Open biopsy of lesion of tissue of parietal lobe of brain
	A081	Biopsy of lesion of tissue of frontal lobe of brain
	A082	Biopsy of lesion of tissue of temporal lobe of brain
	A083	Biopsy of lesion of tissue of parietal lobe of brain
	A084	Biopsy of lesion of tissue of occipital lobe of brain
	A088	Other specified other biopsy of lesion of tissue of brain
