# Supplementary figures and images for: Estimating progression-free survival in patients with glioblastoma using routinely collected data
Source: J Neurooncol. 2017 Sep 27;135(3):621–7. doi: 10.1007/s11060-017-2619-1 (PMC5700233; doi:10.1007/s11060-017-2619-1)

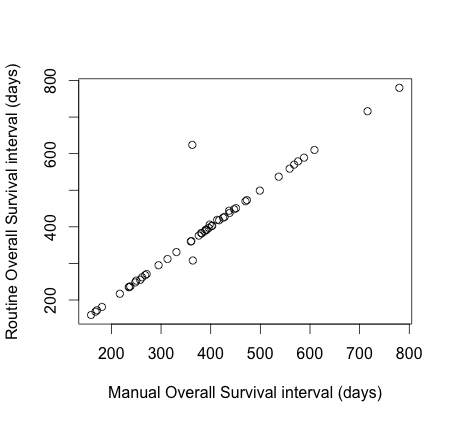

Supplement: Supplementary file 1 — Figure S1. Manual vs. Routine Overall Survival Interval (TIFF 747 KB) [file 11060_2017_2619_MOESM1_ESM.tiff]

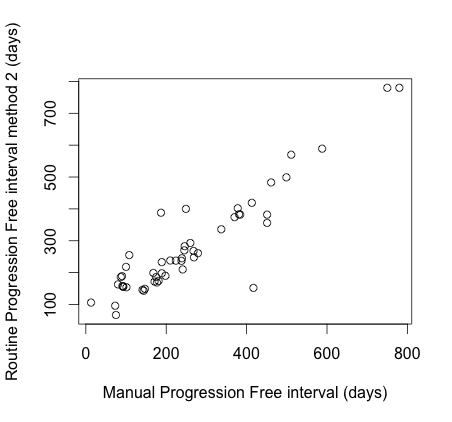

Supplement: Supplementary file 2 — Figure S2 Manual vs. Routine Progression Free Interval (method 1) (TIFF 747 KB) [file 11060_2017_2619_MOESM2_ESM.tiff]

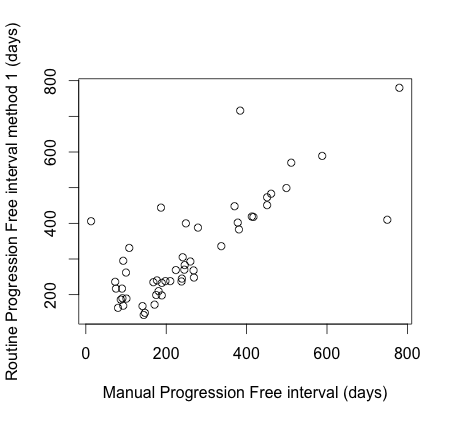

Supplement: Supplementary file 3 — Figure S3 Manual vs. Routine Progression Free Interval (method 2) (TIFF 747 KB) [file 11060_2017_2619_MOESM3_ESM.tiff]
