# Supplementary material for: Estimating progression-free survival in patients with glioblastoma using routinely collected data
Source: J Neurooncol. 2017 Sep 27;135(3):621–7. doi: 10.1007/s11060-017-2619-1 (PMC5700233; doi:10.1007/s11060-017-2619-1)
Supplement: Supplementary file 4 — Table 1. Patient Characteristics (DOC 26 KB) [file 11060_2017_2619_MOESM4_ESM.doc]

Table 1.

| **Age**  Median (and Range) 57 (27-77)  **Sex**  Male 30  Female 20  **Surgical resection** 45  Sub-total resection 29  Gross Total Resection 16  **Biopsy** 5  **MGMT**  Methylated 29  Unmethylated 21 |
| --- |
