# Supplementary material for: Estimating progression-free survival in patients with glioblastoma using routinely collected data
Source: J Neurooncol. 2017 Sep 27;135(3):621–7. doi: 10.1007/s11060-017-2619-1 (PMC5700233; doi:10.1007/s11060-017-2619-1)
Supplement: Supplementary file 5 — Table 2. Sensitivity, specificity and predictive value (DOC 28 KB) [file 11060_2017_2619_MOESM5_ESM.doc]

Table 3.

|  | | **Progression of Glioblastoma** | |  |
| --- | --- | --- | --- | --- |
|  |  | **Yes** | **No** |  |
| **Estimated progression (method 2) 0.75 - 1.25 of Manual PFS** | **Yes** | 23 | 3 | 26 |
|  | **No** | 17 | 7 | 14 |
|  | | 40 | 10 |  |
