# Supplementary material for: Estimating progression-free survival in patients with glioblastoma using routinely collected data
Source: J Neurooncol. 2017 Sep 27;135(3):621–7. doi: 10.1007/s11060-017-2619-1 (PMC5700233; doi:10.1007/s11060-017-2619-1)
Supplement: Supplementary file 6 — Table 3. Acceptable interval sensitivity, specificity, and predictive value (DOC 28 KB) [file 11060_2017_2619_MOESM6_ESM.doc]

Table 2.

|  | | **Progression of Glioblastoma (manual dataset)** | |  |
| --- | --- | --- | --- | --- |
|  |  | **Yes** | **No** |  |
| **Estimated progression using method 2** | **Yes** | 37 | 3 | 40 |
|  | **No** | 3 | 7 | 10 |
|  | | 40 | 10 |  |
